# Epidemiology and Molecular Profiles of ESBL-Producing *Klebsiella pneumoniae* in Urinary Tract Infections Across Jordanian Hospitals

**DOI:** 10.3390/microorganisms14051142

**Published:** 2026-05-19

**Authors:** Ayman Alsheikh, Raghad Shanabla, Ahmad Badawi, Hafez Al-Momani, Mohammed Nasser-Ali, Yaqeen Rjoub, Mohammad A. A. Al-Najjar, Montasir Al-Mansi, Iman Aolymat, Lana Al-Shoubaki, Nawal Al-Zaa’q

**Affiliations:** 1 Department of Medical Laboratory Science, Faculty of Allied Medical Sciences, Zarqa University, P.O. Box 2000, Zarqa 13110, Jordan; raghadshanabla123@gmail.com (R.S.); abadawi@zu.edu.jo (A.B.); mali@zu.edu.jo (M.N.-A.); lalshoubaki@zu.edu.jo (L.A.-S.); nawalzeaq156@gmail.com (N.A.-Z.); 2 Department of Microbiology, Pathology and Forensic Medicine, Faculty of Medicine, The Hashemite University, P.O. Box 330127, Zarqa 13133, Jordan; hafez@hu.edu.jo; 3 Department of Medical Laboratory Sciences, Faculty of Allied Medical Science, Al-Albayt University, P.O. Box 13004, Mafraq 25113, Jordan; yaqeenrjoub99@gmail.com; 4 Faculty of Pharmacy, Applied Science Private University, P.O. Box 541350, Amman 11937, Jordan; moh_alnajjar@asu.edu.jo; 5 Department of Basic Medical Sciences, Faculty of Medicine, Aqaba Medical Sciences University (AMSU), P.O. Box 77110, Aqaba 77110, Jordan; m.mansi@amsu.edu.jo; 6 Department of Anatomy, Physiology and Biochemistry, Faculty of Medicine, The Hashemite University, P.O. Box 330127, Zarqa 13133, Jordan; imank@hu.edu.jo

**Keywords:** antimicrobial resistance, extended-spectrum beta-lactamase, multidrug resistance, *bla*OXA gene, *bla*CTX-M gene, polymerase chain reaction

## Abstract

*Klebsiella pneumoniae* is an opportunistic pathogen associated with both community-acquired and nosocomial infections. Multidrug-resistant (MDR) strains are increasingly implicated in urinary tract infections (UTIs), traveller’s diarrhoea, bacteraemia, and sepsis. β-lactam antibiotics are commonly used for treatment; however, antimicrobial resistance has emerged largely due to the production of extended-spectrum β-lactamases (ESBLs), which confer resistance mainly to penicillins, oxyimino-cephalosporins, and monobactams, while cephamycins and carbapenems usually remain stable to ESBL-mediated hydrolysis and compromise therapeutic efficacy. ESBL-producing strains represent a major cause of severe Gram-negative infections. This study aimed to determine the prevalence of ESBL-producing *K. pneumoniae* among UTI patients in Jordanian hospitals (Al Mafraq, Ma’an, and Islamic Hospitals), evaluate their antimicrobial susceptibility patterns, and detect antimicrobial resistance genes at the molecular level. A total of 450 urine isolates of *K. pneumoniae* were collected from UTI patients between November 2023 and May 2024. Isolates were identified in hospital laboratories using standard microbiological methods. Antimicrobial susceptibility testing was performed, and molecular characterisation of ESBL-associated genes was conducted using polymerase chain reaction (PCR). Out of 450 *K. pneumoniae* isolates collected from UTI patients across three Jordanian regions, 72 (16%) were confirmed as ESBL producers. Among the 72 ESBL-positive *K. pneumoniae* isolates, 34 (47.2%) were recovered from the Central region, 20 (27.8%) from the North, and 18 (25.0%) from the South. Molecular analysis revealed that 41.7% of ESBL-producing isolates carried the *bla*CTX-M gene, while 33.3% harboured the *bla*OXA gene. All ESBL-producing isolates demonstrated antimicrobial resistance to third-generation cephalosporins. A significantly higher proportion of ESBL-producing isolates was identified in female patients (84.7%) compared with males (15.3%). A significant association was observed between *bla*OXA gene distribution and geographic region (*p* = 0.016), whereas *bla*CTX-M gene distribution showed no significant regional association. ESBL-producing *K. pneumoniae* accounted for a substantial proportion of UTI isolates in Jordan, with *bla*CTX-M identified as the predominant resistance gene. The higher burden observed in the Central region and among female patients highlights notable distribution patterns in this cohort. These findings emphasise the necessity for sustained molecular surveillance and strengthened antimicrobial stewardship strategies to limit the dissemination of ESBL-producing strains in Jordanian healthcare settings.

## 1. Introduction

Urinary tract infections (UTIs) are among the most common bacterial infections worldwide and represent a substantial public health burden. It is estimated that approximately 150 million people develop UTIs each year, making them one of the leading causes of healthcare visits and antimicrobial use globally [1]. UTIs result from the invasion and multiplication of pathogenic microorganisms within the urinary tract, most commonly following bacterial entry through the urethra into the bladder. Although a wide range of uropathogens may be involved, *Escherichia coli* remains the predominant causative organism, followed by *Klebsiella pneumoniae* and other Gram-negative bacteria. UTIs occur far more frequently in females than in males, largely because of anatomical and physiological factors, including a shorter urethra, proximity of the urethral opening to the anal orifice, and the absence of prostatic secretions, all of which facilitate ascending infection [1].

Among UTI-associated pathogens, *K. pneumoniae* has emerged as an important opportunistic organism of major clinical concern. This Gram-negative bacterium is widely distributed and is associated with both community-acquired and healthcare-associated infections, including UTIs, pneumonia, bacteraemia, liver abscess, and other invasive infections, particularly among patients with underlying comorbidities [2]. Over the past two decades, *K. pneumoniae* has become the second most common aetiological agent of community-acquired UTIs after *E. coli*. Its significance has been further amplified by its growing role in antimicrobial resistance, prompting the World Health Organization to include it among priority antibiotic-resistant pathogens of international concern [3].

A major contributor to the clinical significance of *K. pneumoniae* is its ability to produce extended-spectrum β-lactamases (ESBLs), enzymes that hydrolyse penicillins, extended-spectrum cephalosporins, and aztreonam, thereby markedly reducing the efficacy of commonly used β-lactam antibiotics [4,5,6]. ESBL-producing *K. pneumoniae* has become a major cause of β-lactam resistance among Gram-negative bacilli in both hospital and community settings [5,7,8]. The spread of ESBL-producing organisms presents serious therapeutic challenges, as these infections are associated with prolonged hospitalisation, increased healthcare costs, limited treatment options, and poorer clinical outcomes [9,10,11].

The genetic basis of ESBL-mediated resistance is often plasmid-borne, which facilitates horizontal transfer of resistance determinants between bacterial species and accelerates the dissemination of multidrug resistance. The most common ESBL-encoding genes include *bla*TEM, *bla*SHV, and *bla*CTX-M, although other genes such as *bla*OXA also contribute to β-lactam resistance in *K. pneumoniae*. Nearly 300 ESBL variants have been described, reflecting the considerable genetic diversity of these enzymes [12,13]. Historically, TEM- and SHV-derived ESBLs were dominant; however, CTX-M-type enzymes have rapidly emerged as the most prevalent ESBLs in many parts of the world and are now regarded as a major driver of resistance among *Enterobacteriaceae* [4,14,15]. Their rapid dissemination has substantial clinical implications because it narrows treatment options, increases morbidity and mortality, and complicates empirical therapy.

Several regional and international studies have documented the growing epidemiological importance of ESBL-producing *K. pneumoniae*. In Jordan, Al-Sheboul, Al-Madi [16] reported high rates of antimicrobial resistance and frequent detection of *bla*SHV and *bla*CTX-M among ESBL-producing *K. pneumoniae* isolates from clinical samples. Similarly, Gharaibeh, Alyafawi [17] demonstrated a high prevalence of multidrug-resistant and ESBL-producing *K. pneumoniae* isolates in Jordanian patients, in addition to detecting carbapenemase genes and the first report of the mcr-1 gene in the country. Aqel, Giakkoupi [18], Aqel, Findlay [19] further documented the emergence of *bla*OXA-48-like, *bla*NDM-1, and *bla*VIM-4 carbapenemase-producing *K. pneumoniae* isolates in Jordanian hospitals, highlighting the ongoing expansion of clinically relevant resistance determinants. More recently, Swedan, Alabdallah [20] reported high rates of non-susceptibility to aminoglycosides and quinolones among clinical *K. pneumoniae* isolates from northern Jordan, together with considerable genetic diversity and multiple associated resistance genes. Collectively, these studies underscore the rapid evolution of *K. pneumoniae* as a multidrug-resistant nosocomial and community pathogen in Jordan.

Evidence from other settings also supports the increasing prevalence of ESBL-associated resistance in UTI pathogens. Mohammedkheir, Gaafar [21] reported high resistance rates and frequent detection of *bla*TEM, *bla*CTX-M, and *bla*SHV among Gram-negative uropathogens in Sudan, while Pereira, Volcão [22] found that ESBL-producing *Klebsiella* spp. were significantly associated with higher resistance rates than non-ESBL-producing isolates in both inpatient and outpatient UTI settings. Likewise, Ghenea, Zlatian [23] reported a predominance of *bla*CTX-M-15 among resistant *K. pneumoniae* isolates, further emphasising the global rise in CTX-M-type β-lactamases. These findings collectively suggest that ESBL production in *K. pneumoniae* is not only widespread but also epidemiologically dynamic, with important regional variation in prevalence and gene distribution.

Despite the growing body of evidence on antimicrobial resistance in Jordan, data specifically addressing the epidemiology and molecular characteristics of ESBL-producing *K. pneumoniae* causing UTIs across multiple Jordanian regions remain limited. Given the clinical burden of UTIs, the increasing prevalence of ESBL production, and the potential for geographic variation in resistance patterns, there is a need for regionally representative data to support infection control and antimicrobial stewardship strategies.

Therefore, the present study aimed to investigate the epidemiology and molecular profiles of ESBL-producing *K. pneumoniae* isolated from UTI patients across Jordanian hospitals. Specifically, the study sought to determine the prevalence of ESBL-producing isolates, assess their antimicrobial resistance patterns, and identify key ESBL-associated resistance genes, particularly *bla*CTX-M and *bla*OXA. The findings of this study are expected to contribute to a better understanding of the molecular epidemiology of ESBL-producing *K. pneumoniae* in Jordan and to support evidence-based interventions for the control of antimicrobial resistance.

## 2. Material and Methods

### 2.1. Study Design and Sample Collection

This prospective, multicentre, cross-sectional laboratory-based investigation was conducted in three hospitals located in the northern, central, and southern regions of Jordan: Al-Mafraq Governmental Hospital, Islamic Hospital in Amman, and Ma’an Governmental Hospital. The study included 450 non-duplicate clinical isolates of *K. pneumoniae* obtained from fresh midstream urine samples collected from hospitalised patients with urinary tract infections in nephrology departments. Samples were collected over a seven-month period, from November 2023 to May 2024. Ethical approval was granted by the Jordanian Ministry of Health (Approval No. 6001; dated 20 May 2024) and the Institutional Review Board of Zarqa University (IRB/ZU/2023/1).

The study enrolled hospitalised patients aged 6 days to 80 years with a clinical diagnosis of urinary tract infection who attended the nephrology departments of the participating hospitals during the study period. Fresh midstream urine samples were collected from eligible patients. Patients with a history of antibiotic use prior to sample collection were excluded. To avoid duplication, only non-duplicate *K. pneumoniae* isolates were included, and only the first isolate recovered from each patient was analysed.

### 2.2. Clinical Isolate Culture

Clinical isolates were subcultured on MacConkey agar and incubated aerobically at 37 °C for 18–24 h. Identification of *K. pneumoniae* was performed based on colony morphology, lactose fermentation characteristics, and standard biochemical tests subsequently confirmed using the VITEK^®^ 2 automated identification system. In addition, molecular confirmation was performed by PCR targeting ESBL-associated genes (*bla*CTX-M and *bla*OXA).

### 2.3. Antimicrobial Susceptibility Testing

Antimicrobial susceptibility testing was performed using the modified Kirby–Bauer disc diffusion method on Mueller–Hinton agar, in accordance with Clinical and Laboratory Standards Institute (CLSI) guidelines [24].

A bacterial suspension equivalent to 0.5 McFarland turbidity standard was prepared. The turbidity was verified using a spectrophotometer at 625 nm to ensure accurate standardization before inoculation. The standardized suspension was then inoculated onto Mueller–Hinton agar plates using sterile swabs. The following antibiotic discs were tested: Amoxicillin-Clavulanic acid (20/10 μg), Cefotaxime (30 μg), Ceftriaxone (30 μg), Ceftazidime (30 μg), Gentamicin (10 μg), Amikacin (30 μg), Ciprofloxacin (5 μg), Imipenem (10 μg), Amoxicillin (30 μg), Cephalexin (30 μg), Cefuroxime (30 μg), Co-trimoxazole (25 μg), Meropenem (10 μg), and Doxycycline (30 μg). The antimicrobial disks used in this study were obtained from Oxoid Ltd., Basingstoke, UK. After incubation at 37 °C for 18–24 h, inhibition zone diameters were measured and interpreted as susceptible, intermediate, or resistant according to CLSI breakpoints.

Quality control for disk diffusion susceptibility testing was performed using *Escherichia coli* ATCC 25922, in accordance with CLSI recommendations. For ESBL-related phenotypic testing, *Klebsiella pneumoniae* ATCC 700603 was used as a supplemental quality control strain.

### 2.4. ESBL Phenotypic Identification by Double Disk Synergy Test

ESBL production was confirmed using the double-disc synergy test (DDST). A lawn culture of *K. pneumoniae* was prepared on Mueller–Hinton agar. An amoxicillin–clavulanic acid disc (20/10 μg) was placed at the centre of the plate, and cefotaxime (30 μg), ceftriaxone (30 μg), and ceftazidime (30 μg) discs were positioned 20 mm apart edge-to-edge, which corresponds approximately to standard centre-to-centre spacing recommended by CLSI [24].

Following incubation at 37 °C for 18–24 h, a ≥5 mm increase in the zone of inhibition towards the clavulanic acid-containing disc indicated ESBL production.

*K. pneumoniae* ATCC 700603 was used as the positive control, and *Escherichia coli* ATCC 25922 served as the negative control.

Prepared CHROM agar ESBL plates (CHROM agar, Paris, France) were used for the phenotypic detection of ESBL-producing isolates. Each bacterial strain was inoculated onto CHROM agar ESBL and incubated aerobically at 37 °C for 18–24 h. ESBL-producing colonies displayed species-specific colours, with *E. coli* appearing dark pink to reddish and *Klebsiella* spp. appearing metallic blue. In contrast, non-ESBL-producing isolates produced colourless colonies or showed no growth on CHROM agar ESBL [5].

### 2.5. DNA Extractions

Genomic DNA was extracted from cultured isolates by alkaline lysis, as previously described [5,25]. Briefly, a single bacterial colony was suspended in 20 μL of lysis buffer (0.25% sodium dodecyl sulfate, 0.05 N NaOH) and heated at 95 °C for 15 min. The lysate was then diluted with 180 μL of sterile distilled water and centrifuged at 16,000× *g* for 5 min to remove residual debris. The resulting supernatant was collected and used as the DNA template for polymerase chain reaction (PCR) or stored at −20 °C until further analysis. DNA concentration and purity were measured using a PG/T60 UV–visible spectrophotometer (PG Instruments Limited, Beijing, China). DNA quality was confirmed by successful downstream amplification.

Genomic DNA was extracted using the GF-1 Bacterial DNA Extraction Kit (Vivantis Technologies, Subang Jaya, Malaysia) according to the manufacturer’s instructions. DNA was eluted according to the kit protocol.

### 2.6. Polymerase Chain Reaction (PCR)

PCR amplification of *bla*OXA and *bla*CTX-M genes was performed using a Veriti Thermal Cycler (Applied Biosystems, San Francisco, CA, USA). Primers (10 μM) were synthesised by a commercial oligonucleotide manufacturer (sequences shown in Table 1).

For detection of the blaOXA gene, each PCR was carried out in a total volume of 20 µL, containing 2 µL of extracted DNA, 0.6 µL of forward primer, 0.6 µL of reverse primer, 4 µL of master mix, and 12.8 µL of nuclease-free water (NFW). The amplification conditions consisted of 30 cycles of denaturation at 95 °C for 30 s, annealing at 57 °C for 30 s, and extension at 72 °C for 30 s.

For amplification of the blaCTX-M gene, PCR was performed under similar conditions using forward and reverse primers (10 µM; SOLIS BIODYNE, FIREPol^®^, Helsinki, Finland). Each 20 µL reaction mixture contained 2 µL of extracted DNA, 0.6 µL of each primer, 4 µL of master mix, and 12.8 µL of nuclease-free water. The cycling conditions included 30 cycles of denaturation at 95 °C for 30 s, annealing at 60 °C for 30 s, and extension at 72 °C for 30 s.

A previously characterised clinical isolate known to harbour the target resistance gene was included as positive control in each PCR run. A *bla*CTX-M-positive *K. pneumoniae* isolate was used as the positive control for *bla*CTX-M amplification, while a sequencing-confirmed *bla*OXA-positive isolate was used as the positive control for *bla*OXA amplification. Nuclease-free water was included as a negative control.

### 2.7. Gel Electrophoresis

PCR products were analysed using electrophoresis on 1.5% low-melting agarose gel (Vivantis, Shah Alam, Malaysia), which is suitable for resolving fragments in the 550–816 bp range. Electrophoresis was conducted at 100 V for 50 min using an electrophoresis apparatus (Thistle Scientific Ltd., Edinburgh, UK).

DNA bands were visualised under a UV transilluminator. Both 100 bp and 1000 bp DNA ladder (GeneDireX, Taoyuan City, Taiwan) were used to accurately estimate fragment sizes, with the 100 bp ladder for smaller fragments and the 1000 bp ladder for larger fragments.

### 2.8. Statistical Analysis

Statistical analysis was performed using IBM SPSS Statistics version 23 (IBM Corp., Armonk, NY, USA). Descriptive statistics were used to summarise the distribution of ESBL-producing *K. pneumoniae* isolates and resistance genes across study variables. Frequencies and percentages were calculated for categorical variables, while means and standard deviations were generated for numerical or coded variables. Differences between two groups were analysed using the independent-samples *t*-test, whereas comparisons among multiple groups were assessed using one-way ANOVA, followed by Tukey and Duncan post hoc tests when appropriate. Associations between categorical variables were evaluated using the chi-square test or Fisher’s exact test where required. Correlations were assessed using Pearson’s and Spearman’s correlation coefficients. Statistical significance was set at *p* < 0.05.

## 3. Result

### 3.1. Prevalence of ESBL-Producing Klebsiella pneumoniae

A total of 450 *K. pneumoniae* isolates were collected using a quota sampling approach from patients with urinary tract infections across regions (150 isolates per region: North, Central, and South) between November 2023 and May 2024. Among these isolates, 72 (16%) were confirmed as ESBL producers (Table 2). Variation in the distribution of ESBL-producing isolates was observed across hospitals. Among the 72 ESBL-producing isolates, 34 (47.2%) were recovered from the Islamic Hospital in the Central region, 20 (27.8%) from Al-Mafraq Governmental Hospital in the North, and 18 (25.0%) from Ma’an Governmental Hospital in the South (Table 3). ESBL-producing isolates were recovered from patients of both sexes, with ages ranging from 6 days to 80 years, and were distributed across all predefined age groups, with the highest proportion observed among patients aged 51–80 years.

### 3.2. Modified Kirby–Bauer Disc Diffusion Results

The modified Kirby–Bauer disc diffusion test showed that 72 of the 450 isolates were resistant to third-generation cephalosporins, including cefotaxime, ceftriaxone, and ceftazidime, relative to the other antibiotics tested. According to the inhibition zone diameters, the isolates were categorised as resistant, intermediate, or susceptible (Supplementary Figure S1).

### 3.3. Phenotypic Confirmation of ESBL Production

Phenotypic confirmation using the double-disc synergy test (DDST) demonstrated that all 72 ESBL-producing isolates were resistant to third-generation cephalosporins and exhibited synergy between amoxicillin–clavulanic acid and at least one cephalosporin disc, confirming ESBL production (Supplementary Figure S2).

### 3.4. Distribution of ESBL-Associated Genes

#### 3.4.1. blaOXA Gene Distribution

Among the 72 ESBL-producing isolates, 24 (33.3%) were positive for the *bla*OXA gene, while 48 (66.7%) were negative (Table 4). Molecular analysis detected the *bla*CTX-M gene in 41.7% of ESBL-producing isolates and the *bla*OXA gene in 33.3%. While blaCTX-M is a well-recognised ESBL-associated gene, *bla*OXA was interpreted in this study more cautiously as a class D β-lactamase gene, because OXA enzymes comprise a diverse group that includes narrow-spectrum β-lactamases, carbapenemases, and, in some variants, enzymes with ESBL-like activity.

**Figure 1:**
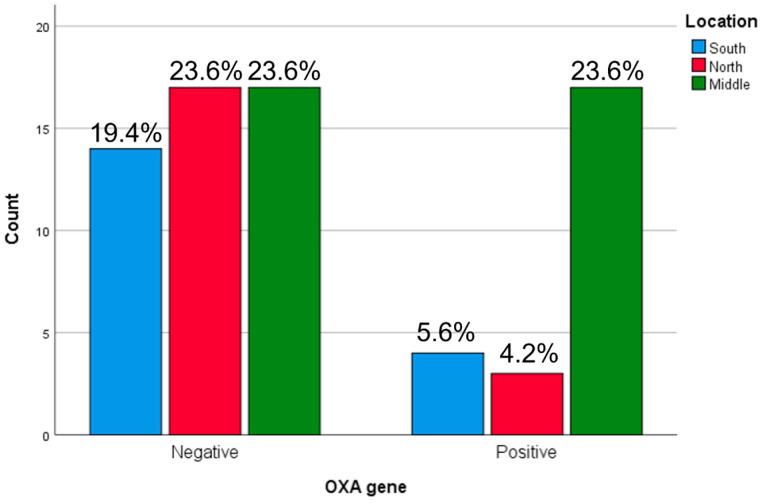
Geographical distribution of the *bla*OXA gene among ESBL-producing *K. pneumoniae* isolates. OXA-positive isolates were observed in the Central regions (23.6%) and Southern regions (5.6%) while the Northern regions showed the lowest proportion (4.2%).

Geographically, the highest proportion of OXA-positive isolates was observed in the Central regions (23.6%) and Southern regions (5.6%) while the Northern regions showed the lowest proportion (4.2%). A statistically significant association was found between OXA gene distribution and geographic region (*p* = 0.016) (Figure 1).

**Figure 2:**
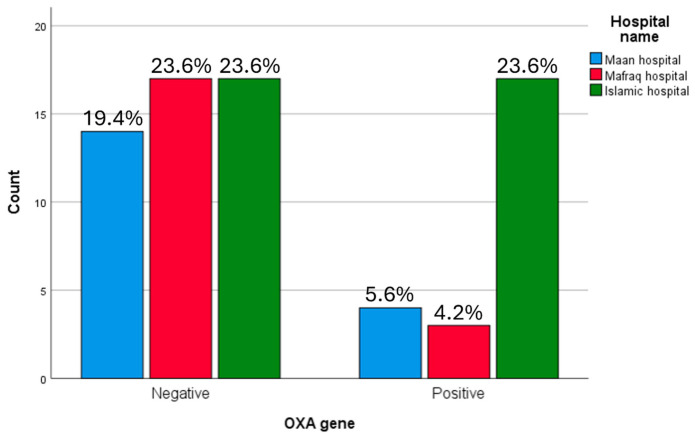
*bla*OXA gene correlation with medical centre in ESBL producing *K. pneumoniae* samples. OXA-positive isolates were observed in the Islamic hospital (23.6%) and Maan hospital (5.6%) while the Mafraq hospital showed the lowest proportion (4.2%).

However, when analysed by individual hospital, no statistically significant association was observed between OXA gene presence and medical centre (*p* = 0.16) (Figure 2).

#### 3.4.2. blaCTX-M Gene Distribution

The *bla*CTX-M gene was detected in 30 of the 72 ESBL-producing isolates (41.7%), while 42 isolates (58.3%) were negative (Table 5).

**Figure 3:**
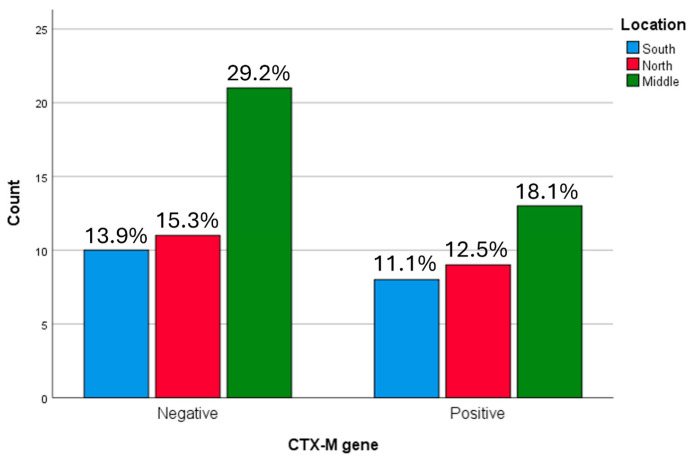
Geographical position affects the *bla*CTX gene in ESBL producing *K. pneumoniae* samples. The highest proportion of CTX-M-positive isolates was observed in the Middle region (18.1%).

**Figure 4:**
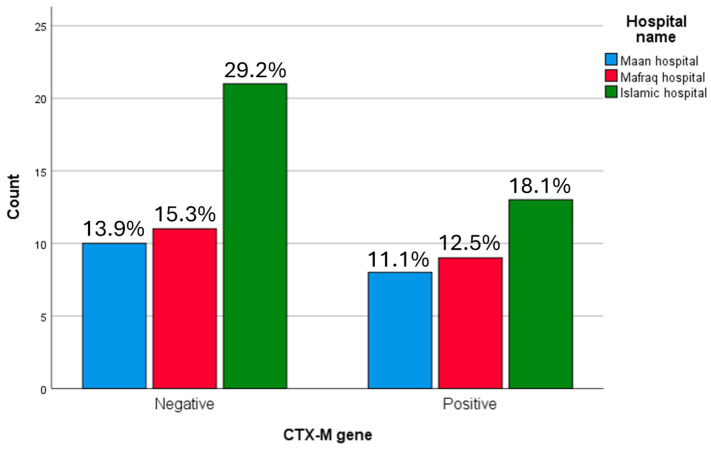
*bla*CTX gene correlation with medical centre in ESBL producing *K. pneumoniae* samples. The highest proportion of CTX-M-positive isolates was observed in the Islamic hospital (18.1%).

The highest proportion of *bla*CTX-M-positive isolates was observed in the Central region (18.1%). No statistically significant association was identified between *bla*CTX-M gene distribution and geographic region (*p* = 0.855) (Figure 3), nor between *bla*CTX-M distribution and individual hospital (*p* = 0.855) (Figure 4).

#### 3.4.3. Gender Distribution

Among ESBL-producing isolates: 84.7% (*n* = 61) were recovered from female patients and 15.3% (*n* = 11) were recovered from male patients (Table 6). This higher number of isolates in females likely reflects the naturally higher prevalence of urinary tract infections in females rather than a true epidemiological difference.

**Figure 5:**
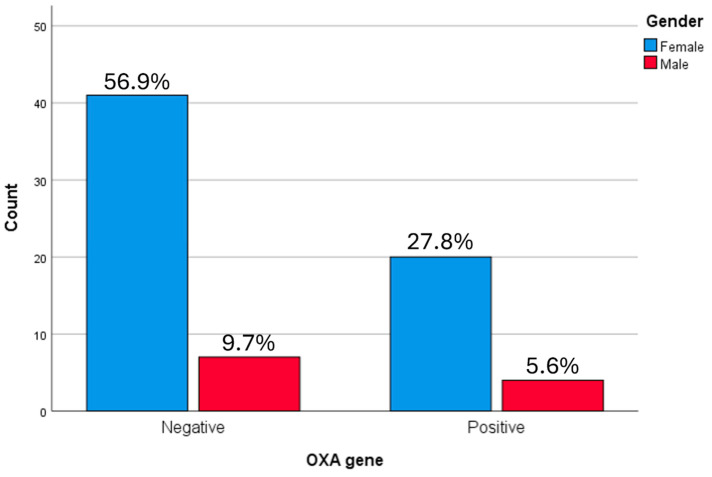
Relationship between the categories of Gender and the *bla*OXA gene in ESBL producing *K. pneumoniae* samples. Females showed a higher absolute frequency of both OXA and CTX-M genes (27.8%); however, no statistically significant association was found between OXA gene presence and gender as showed by ANOVA test.

Although females showed a higher absolute frequency of both *bla*OXA and *bla*CTX-M genes, no statistically significant association was found between *bla*OXA gene presence and gender (*p* = 0.817) as shown by ANOVA test (Figure 5).

**Figure 6:**
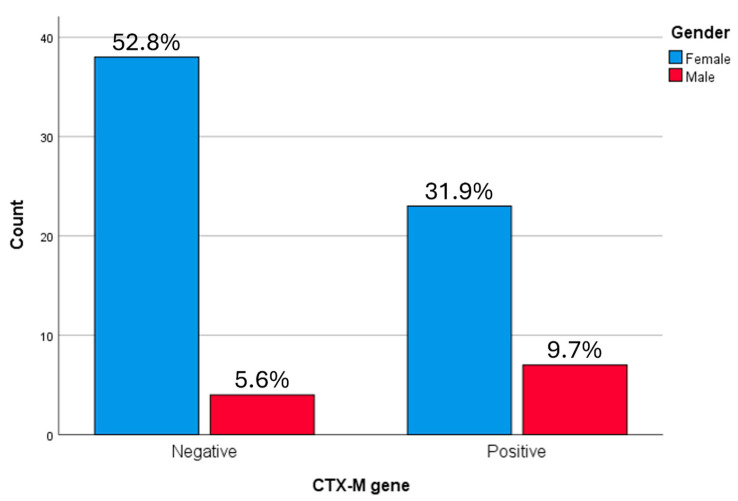
Relationship between the categories of Gender and the *bla*CTX gene in ESBL producing *K. pneumoniae* samples. A statistically significant association was observed between CTX-M gene distribution and gender as showed by *t*-test analysis, with females demonstrating a higher proportion (31.9%) of CTX-M-positive isolates.

A statistically significant association was observed between *bla*CTX-M gene distribution and gender (*p* = 0.0108) as shown by t-test analysis (Figure 6), with females demonstrating a higher proportion of *bla*CTX-M-positive isolates.

#### 3.4.4. Age Distribution

ESBL-producing *K. pneumoniae* isolates were distributed across all age groups. The highest proportion was observed in patients aged 51–80 years (41.7%), followed by: 6 days–9 years: 22.2%, 10–30 years: 18.1%, 31–50 years: 18.1%, (Table 7). It should be noted that the age groups are uneven, which may introduce bias in statistical comparisons; therefore, age-related findings should be interpreted with caution.

**Figure 7:**
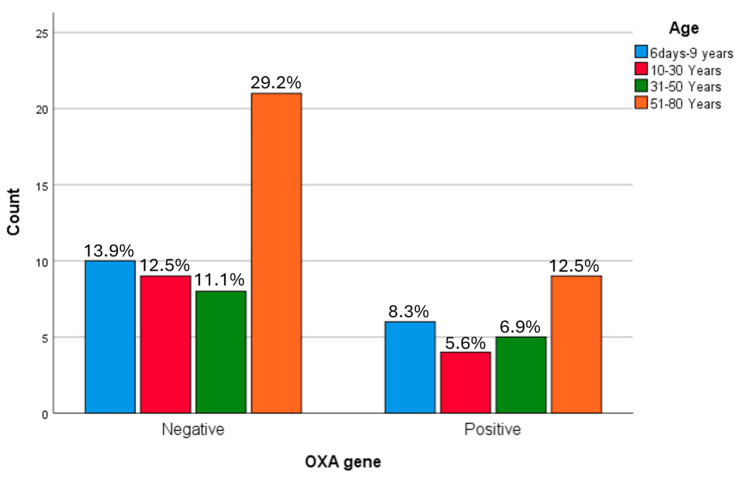
The demographic correlation between the *bla*OXA gene in ESBL producing *K. pneumoniae*. The 51–80-year age group demonstrated the highest proportion of OXA-positive isolates (12.5%), while the lowest proportion was observed in the 10–30-year group (5.6%). No statistically significant association was identified between OXA gene presence and age group.

The 51–80-year age group demonstrated the highest proportion of *bla*OXA-positive isolates (12.5%), while the lowest proportion was observed in the 10–30-year group (5.6%). No statistically significant association was identified between *bla*OXA gene presence and age group (*p* = 0.926) (Figure 7).

**Figure 8:**
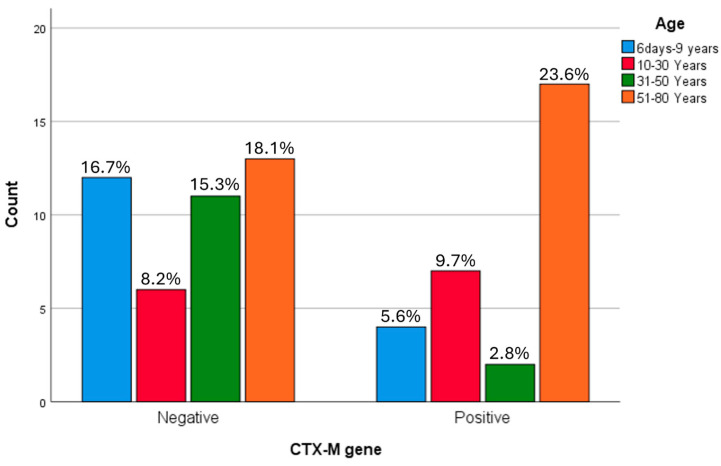
The demographic correlation between the *bla*CTX gene in ESBL producing *K. pneumoniae*. the highest frequency of *bla*CTX-M positivity was observed in the 51–80-year age group; however, no statistically significant association was found between *bla*CTX-M distribution and age.

Similarly, although the highest frequency of *bla*CTX-M positivity was observed in the 51–80-year age group, no statistically significant association was found between *bla*CTX-M distribution and age (Figure 8).

## 4. Discussion

This multicentre study provides insight into the epidemiology and molecular characteristics of ESBL-producing *K. pneumoniae* among patients with urinary tract infections (UTIs) in Jordan. Of the 450 urinary *K. pneumoniae* isolates examined, 72 (16%) were confirmed as ESBL producers, indicating that ESBL-mediated resistance represents a substantial proportion of *K. pneumoniae*-associated UTIs in the participating hospitals. This finding is clinically important because ESBL production compromises the activity of key β-lactam agents, particularly oxyimino-cephalosporins such as cefotaxime, ceftriaxone, and ceftazidime, thereby limiting therapeutic options and increasing the risk of treatment failure [27,28]. The growing burden of ESBL-producing Gram-negative bacteria in both healthcare and community settings has been recognised as a major therapeutic and public health challenge worldwide [7,10,11].

All ESBL-producing isolates in the present study were resistant to third-generation cephalosporins, which is consistent with the recognised phenotype of ESBL-producing Enterobacterales. ESBL enzymes hydrolyse penicillins, aztreonam, and extended-spectrum cephalosporins, rendering these antibiotics ineffective and complicating empirical treatment strategies [4,6,28]. This pattern suggests that resistance in the current isolates is not sporadic, but more likely reflects sustained antimicrobial selection pressure in clinical settings. Inappropriate antibiotic use, overuse of broad-spectrum antimicrobials, and inadequate stewardship are well-established drivers of such resistance [29,30]. In this context, the present findings reinforce the need for routine phenotypic detection of ESBLs in hospital microbiology laboratories and for more judicious use of cephalosporins in UTI management.

At the molecular level, *bla*CTX-M was detected more frequently than *bla*OXA among ESBL-producing isolates, indicating that CTX-M-type enzymes are likely to be the dominant ESBL determinants in this setting. This observation is consistent with previous studies showing the global emergence and rapid dissemination of CTX-M enzymes among Enterobacterales [4,14,15]. CTX-M β-lactamases have increasingly replaced TEM- and SHV-derived ESBLs in many parts of the world and are now regarded as the predominant non-TEM, non-SHV ESBL family [12,13]. Their epidemiological success is largely attributable to plasmid-mediated transmission, which facilitates rapid intra- and inter-species spread of resistance genes [27]. Therefore, the predominance of *bla*CTX-M in the present study may reflect the broader global shift towards CTX-M-mediated resistance and suggests that transferable resistance elements are likely contributing to the local dissemination of ESBL-producing *K. pneumoniae*.

The detection of *bla*OXA in one-third of ESBL-producing isolates is also noteworthy. Although *bla*OXA is more commonly discussed in relation to carbapenem resistance, OXA-type β-lactamases have also been reported in multidrug-resistant *K. pneumoniae* and may coexist with other resistance determinants, thereby contributing to more complex resistance phenotypes [18,19]. The coexistence of multiple β-lactamase genes within the same bacterial background has important clinical implications, as it may enhance resistance breadth and reduce the effectiveness of standard treatment regimens [31]. While only *bla*CTX-M and *bla*OXA were investigated in the current study, the observed resistance profile suggests that additional ESBL-associated genes, such as *bla*SHV and *bla*TEM, may also be present but were not captured in the current analysis. This interpretation is supported by prior studies from Jordan and elsewhere, which documented frequent detection of *bla*SHV, *bla*CTX-M, and *bla*TEM among clinical *K. pneumoniae* isolates [16,21].

The regional distribution of ESBL-producing isolates in this study showed the highest proportion in the central region, followed by the north and south. Although these data are descriptive, they may reflect differences in population density, healthcare utilisation, referral patterns, hospital case complexity, and antimicrobial exposure. Urban and central hospitals often receive a larger and more clinically complex patient population, which may increase opportunities for antimicrobial selection pressure and nosocomial transmission. Similar geographic variation in resistance burden has been observed in other settings and has been linked to disparities in prescribing practices, infection control infrastructure, and antibiotic accessibility [23,32]. Moreover, the significant association observed between *bla*OXA distribution and geographic region in the present study suggests that local ecological or healthcare-related factors may influence the circulation of specific resistance determinants. Although geographic and gender differences were observed, the present study was not designed to determine the factors underlying these distributions. In the absence of data on prior antibiotic exposure, healthcare-associated risk factors, catheter use, and comorbidities, no causal inference can be made regarding the observed patterns.

By contrast, no consistent statistically significant associations were found between most resistance genes and demographic variables such as age, sex, hospital, or region. These negative findings are still informative. They suggest that once ESBL-producing *K. pneumoniae* becomes established within a healthcare environment, resistance genes may spread across a broad patient population rather than remaining confined to a specific subgroup. Such dissemination is compatible with plasmid-mediated transmission and with the broad circulation of resistant strains in both hospital and community interfaces [27,29]. Nevertheless, the lack of statistical significance should be interpreted cautiously, as the number of ESBL-producing isolates available for subgroup analysis may have limited the power to detect modest associations.

The pronounced predominance of female patients among ESBL-producing UTI cases in the present study is in keeping with the established epidemiology of UTIs. Women are known to be more susceptible to UTIs because of anatomical and physiological factors, including a shorter urethra, proximity of the urethral opening to the anal orifice, hormonal influences, and certain reproductive or sexual health-related exposures [1,33]. The higher proportion of female cases observed here therefore most likely reflects the greater baseline burden of UTI in women, rather than a sex-specific tendency to acquire ESBL genes. This distinction is important, as it suggests that female predominance in the present cohort should be interpreted as a feature of disease epidemiology rather than evidence of a different molecular resistance mechanism.

Similarly, the greater frequency of ESBL-producing isolates in older patients is clinically plausible. Advanced age is often associated with increased healthcare contact, repeated antibiotic exposure, urinary catheterisation, chronic comorbidities, and recurrent or complicated UTIs, all of which can increase the risk of infection with resistant organisms. Previous studies have also reported greater UTI burden and higher frequencies of resistant isolates among older adults [21]. Therefore, the higher proportion of ESBL-producing *K. pneumoniae* in older age groups in the present study is likely to reflect cumulative exposure to healthcare-associated and antimicrobial-related risk factors rather than age as an isolated biological determinant.

The present findings are broadly consistent with previous studies from Jordan and other regions. In Jordan, Al-Sheboul, Al-Madi [16] reported high rates of ESBL-producing *K. pneumoniae* carrying *bla*SHV and *bla*CTX-M, while Gharaibeh, Alyafawi [17] documented a high prevalence of multidrug resistance and ESBL production among clinical *K. pneumoniae* isolates. Aqel, Giakkoupi [18,19] also demonstrated the emergence of clinically significant β-lactamase genes, including *bla*OXA-48-like and *bla*NDM-1, in Jordanian hospitals, underscoring the dynamic and expanding resistance landscape in the country. Internationally, studies from Sudan, Romania, and Brazil have likewise shown that *bla*CTX-M is a frequent determinant among ESBL-producing uropathogens and that ESBL-positive isolates exhibit higher resistance rates across multiple antimicrobial classes [21,22,23]. Taken together, these findings position the current study within a wider pattern of increasing CTX-M-associated resistance among clinically important Enterobacterales.

The clinical implications of these findings are considerable. ESBL-producing *K. pneumoniae* can limit the usefulness of empirical β-lactam therapy and force reliance on broader-spectrum or reserve agents, thereby increasing treatment cost and potentially accelerating resistance selection to last-line drugs. In children and other vulnerable patients, ESBL-associated UTI has been linked to hospitalisation, delayed appropriate therapy, and difficulty in selecting effective prophylactic or empirical treatment [9]. The burden identified in the current study therefore supports the need for regular local surveillance, hospital-specific antibiograms, and stewardship interventions tailored to Jordanian healthcare settings. Because resistance patterns may vary between institutions and regions, locally generated data are essential for guiding empirical treatment policies.

Overall, the present study highlights that ESBL-producing *K. pneumoniae* is an important cause of UTI in Jordanian hospitals, with *bla*CTX-M emerging as the predominant detected ESBL-associated gene and *bla*OXA also contributing to the resistance profile. These findings support the view that plasmid-mediated resistance dissemination, antibiotic selection pressure, and healthcare-associated transmission are likely to be key drivers of the observed patterns. Strengthening laboratory detection capacity, implementing continuous molecular surveillance, and reinforcing antimicrobial stewardship and infection prevention programmes are therefore critical steps in limiting the further spread of ESBL-producing *K. pneumoniae* in Jordan [17,20,29].

This study was limited by its inclusion of only three hospitals and urine isolates from hospitalised UTI patients, which may restrict the generalisability of the findings to other healthcare settings and community-acquired infections. Molecular analysis was confined to *bla*CTX-M and *bla*OXA, and other relevant resistance genes were not investigated. In addition, no sequencing or molecular typing was performed, preventing assessment of clonal relatedness and transmission dynamics. The absence of detailed clinical risk factor data and MIC-based susceptibility testing further limited comprehensive evaluation of resistance determinants and their clinical significance.

### Recommendations

The findings of this study, particularly the high prevalence of *bla*CTX-M-mediated resistance in *K. pneumoniae* isolates from nephrology patients, highlight the urgent need to strengthen laboratory infrastructure and implement standardised protocols for continuous surveillance of antimicrobial resistance, Surveillance strategies should prioritize departments and patient populations showing the highest burden of ESBL-producing isolates, such as nephrology and catheterized patients, as identified in this study. Routine phenotypic and molecular monitoring of resistant isolates should be integrated into hospital diagnostic workflows to enable timely detection and guide targeted antimicrobial therapy based on local resistance patterns observed in this cohort.

In addition, robust antimicrobial stewardship programmes must be reinforced to minimise inappropriate antibiotic prescribing and reduce selection pressure. Educational initiatives targeting healthcare professionals, microbiologists, and the general public are essential to improve awareness of antimicrobial resistance and promote responsible antibiotic use. Furthermore, strict adherence to infection prevention and control measures, including improved hygiene practices within healthcare settings, is imperative to limit the transmission of ESBL-producing strains. This study has some limitations. First, isolates were collected exclusively from nephrology departments, which may introduce selection bias and limit the generalizability of the findings to the broader population of UTI patients.

## 5. Conclusions

This study demonstrates that the *bla*CTX-M gene is the predominant ESBL-associated gene among *K. pneumoniae* isolates recovered from UTI patients in Jordanian hospitals. The detection of both *bla*CTX-M and *bla*OXA antimicrobial resistance determinants confirms the circulation of clinically significant ESBL genotypes across multiple regions of the country.

Moreover, a higher proportion of ESBL-producing *K. pneumoniae* UTIs was observed among female patients compared with males, reflecting known epidemiological susceptibility patterns. These findings underscore the importance of sustained molecular surveillance, targeted antimicrobial stewardship strategies, and strengthened infection control measures to mitigate the spread of ESBL-producing *K. pneumoniae* in Jordan.

## Figures and Tables

**Table 1:** Primer sequences used in this study.

Gene	Primer Sequences	Product Size (bp)	Annealing T and Time	References
*bla*OXA	Forward (5′-GGTTAGTTGGCCCCCTTAAA-3′) Reverse (5′-AGTTGAGCGAAAAGGGGATT-3′)	816 bp	57 °C for 30 s	[26]
*bla*CTX-M	Forward (5′-ACCGCGATATCGTTGGT-3′) Reverse (5′-CGCTTTGCGATGTGCAG-3′)	550 bp	60 °C for 30 s	[15]

**Table 2:** Distribution of ESBL-producing *K. pneumoniae* among total *K. pneumoniae* isolates.

Type	Number of Specimens	Percentage
Negative ESBL	378	84%
Positive ESBL	72	16%
Total	450	100%

**Table 3:** Distribution of ESBL-producing *K. pneumoniae* isolates across healthcare institutions.

Hospital Name	Number of Specimens	Percentage
Maan hospital	18	25.0%
Mafraq hospital	20	27.8%
Islamic hospital	34	47.2%
Total	72	100.0%

**Table 4:** The *bla*OXA gene distribution in *K. pneumoniae* generated for ESBL samples.

	Number of Specimens	Percentage
Negative	48	66.7%
Positive	24	33.3%
Total	72	100.0%

**Table 5:** The *bla*CTX gene distribution in ESBL producing *K. pneumoniae* samples.

	Number of Specimens	Percentage
Negative	42	58.3
Positive	30	41.7
Total	72	100.0

**Table 6:** Distribution of ESBL-producing *K. pneumoniae* isolates by patient gender.

Gender	Number of Specimens	Percentage
Female	61	84.7%
Male	11	15.3%
Total	72	100.0%

**Table 7:** Age distribution of ESBL-producing *K. pneumoniae* isolates.

Age	Number of Specimens	Percentage
6 Days–9 years	16	22.2
10–30 years	13	18.1
31–50 years	13	18.1
51–80 years	30	41.7
Total	72	100.0

## Data Availability

The original contributions presented in this study are included in the article/Supplementary Materials. Further inquiries can be directed to the corresponding author.

## Supplementary Materials

The following supporting information can be downloaded at: https://www.mdpi.com/article/10.3390/microorganisms14051142/s1, Figure S1: Susceptibility patterns of *K. pneumoniae* produces ESBL; Figure S2: The double disk synergy test; Figure S3: The OXA gene band of *K. pneumoniae* generated for the ESBL sample in gel electrophoresis; Figure S4: The OXA gene of *K. pneumoniae* generated for ESBL sample in gel electrophoresis; Table S1: Prevalence and Distribution of OXA and CTX-M Genes in *K. pneumoniae* Isolates from UTI Patients Across Jordan; Table S2: Distribution Patterns of The OXA Gene in *K. pneumoniae* Isolates by Location, Hospital, Gender, and Age; Table S3: Impact of Location, Hospital Name, Gender, And Age on the Distribution of The OXA Gene in *K. pneumoniae* Isolates; Table S4: Assessment of CTX-M Gene Distribution Across Location, Hospital, Name, Gender, and Age; Table S5: Conference Interval Analysis of Mean Differences in CTX-M Gene Distribution by Location, Hospital Name, Gender, and Age; Table S6: Geographical Distribution of OXA Gene Cases Across Regions; Table S7: Geographical Distribution of CTX-M Gene Cases Across Regions.
