# Supplementary material for: Epidemiology and Molecular Profiles of ESBL-Producing Klebsiella pneumoniae in Urinary Tract Infections Across Jordanian Hospitals
Source: Microorganisms. 2026 May 19;14(5):1142. doi: 10.3390/microorganisms14051142 (PMC13209261; doi:10.3390/microorganisms14051142)
Supplement: Supplementary file 1 [file microorganisms-14-01142-s001.zip › microorganisms-4187741-supplementary.pdf]

Supplementary materials

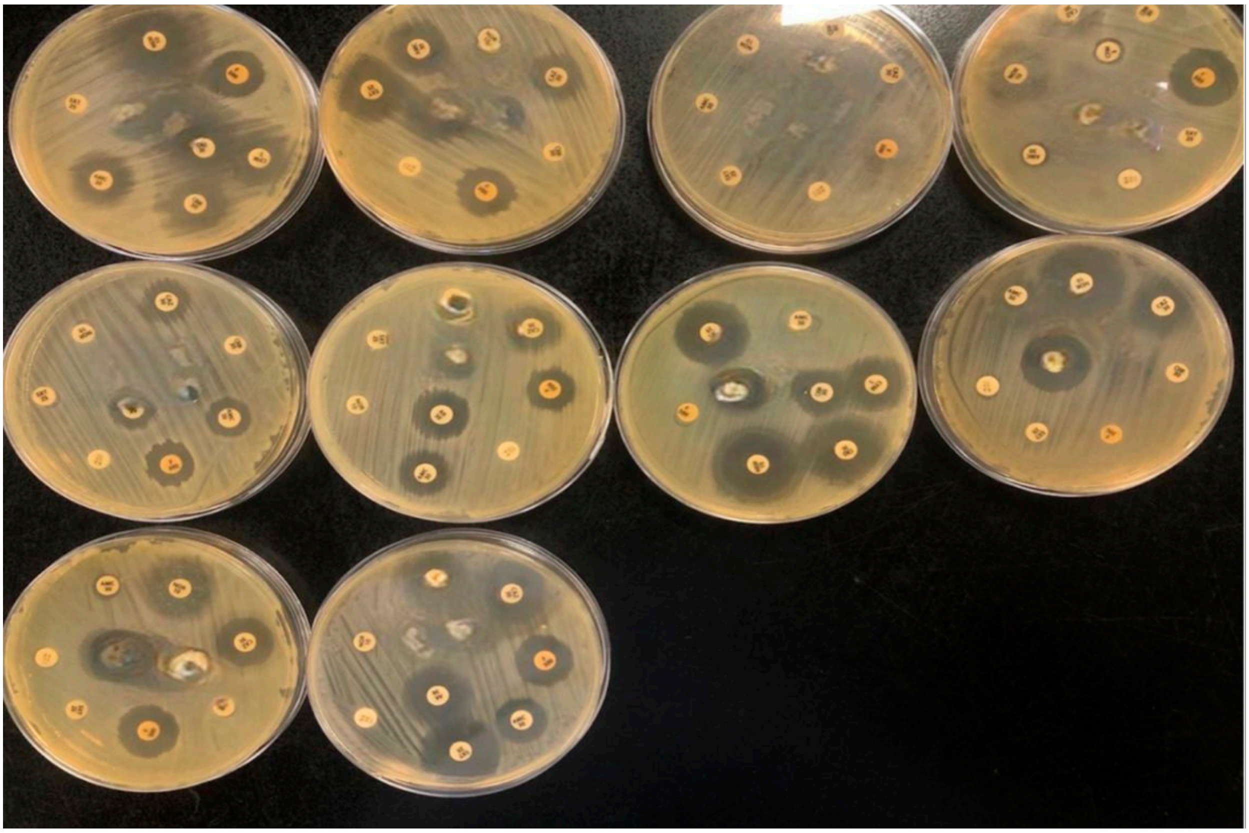

Figure S1: Susceptibility patterns of *K. Pneumoniae* produces ESBL.

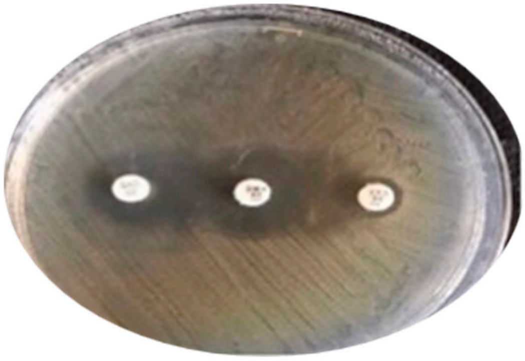

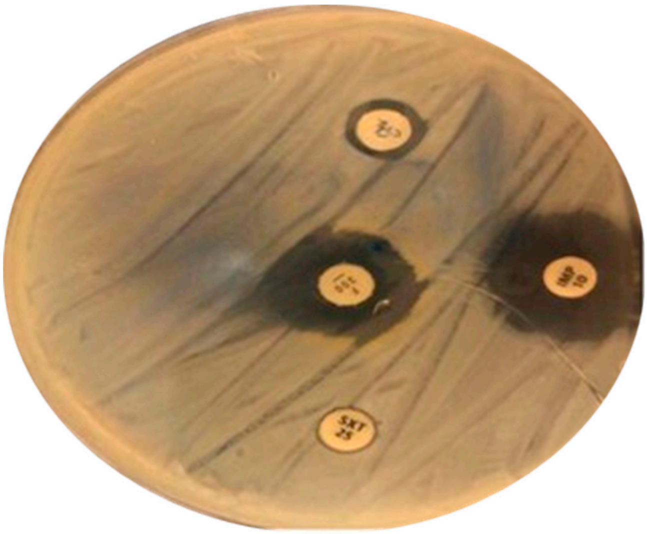

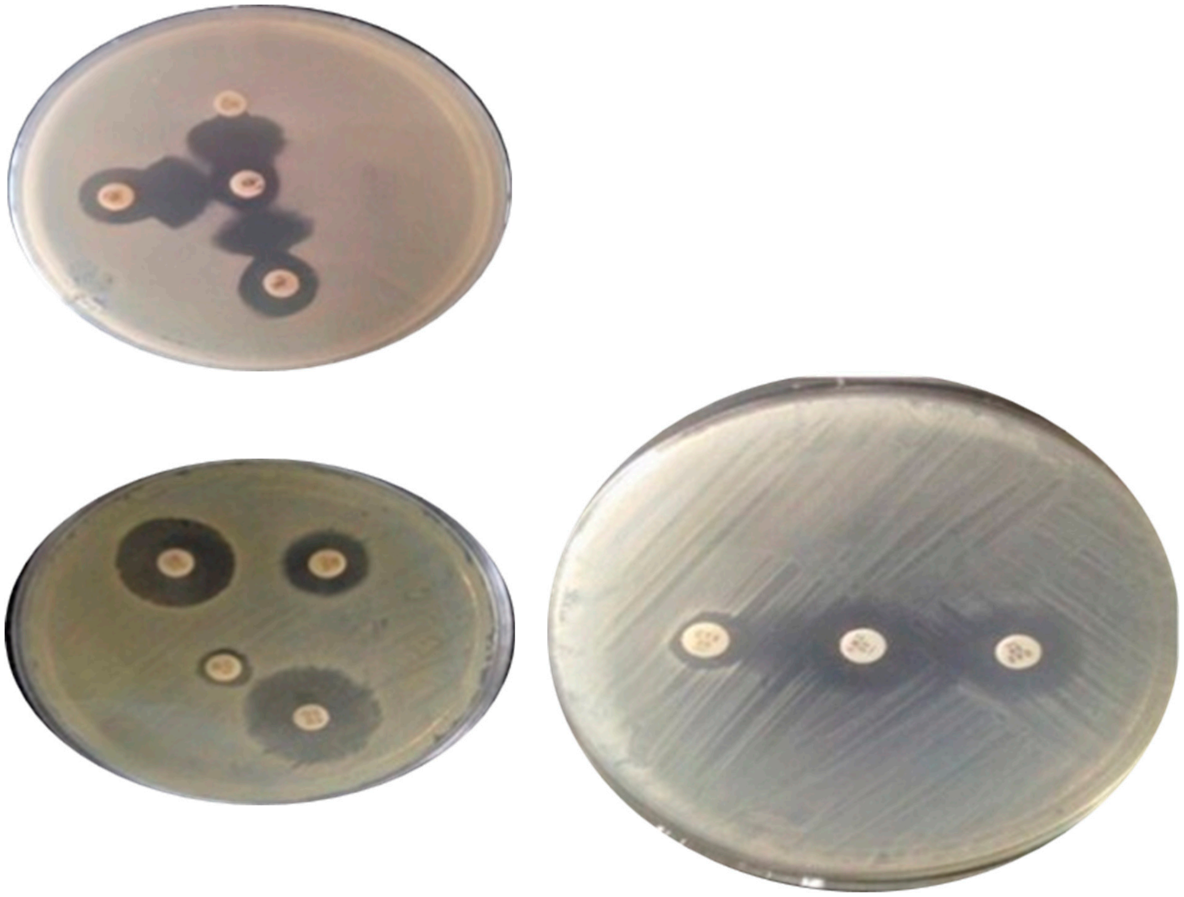

Figure S2: The double disk synergy test.

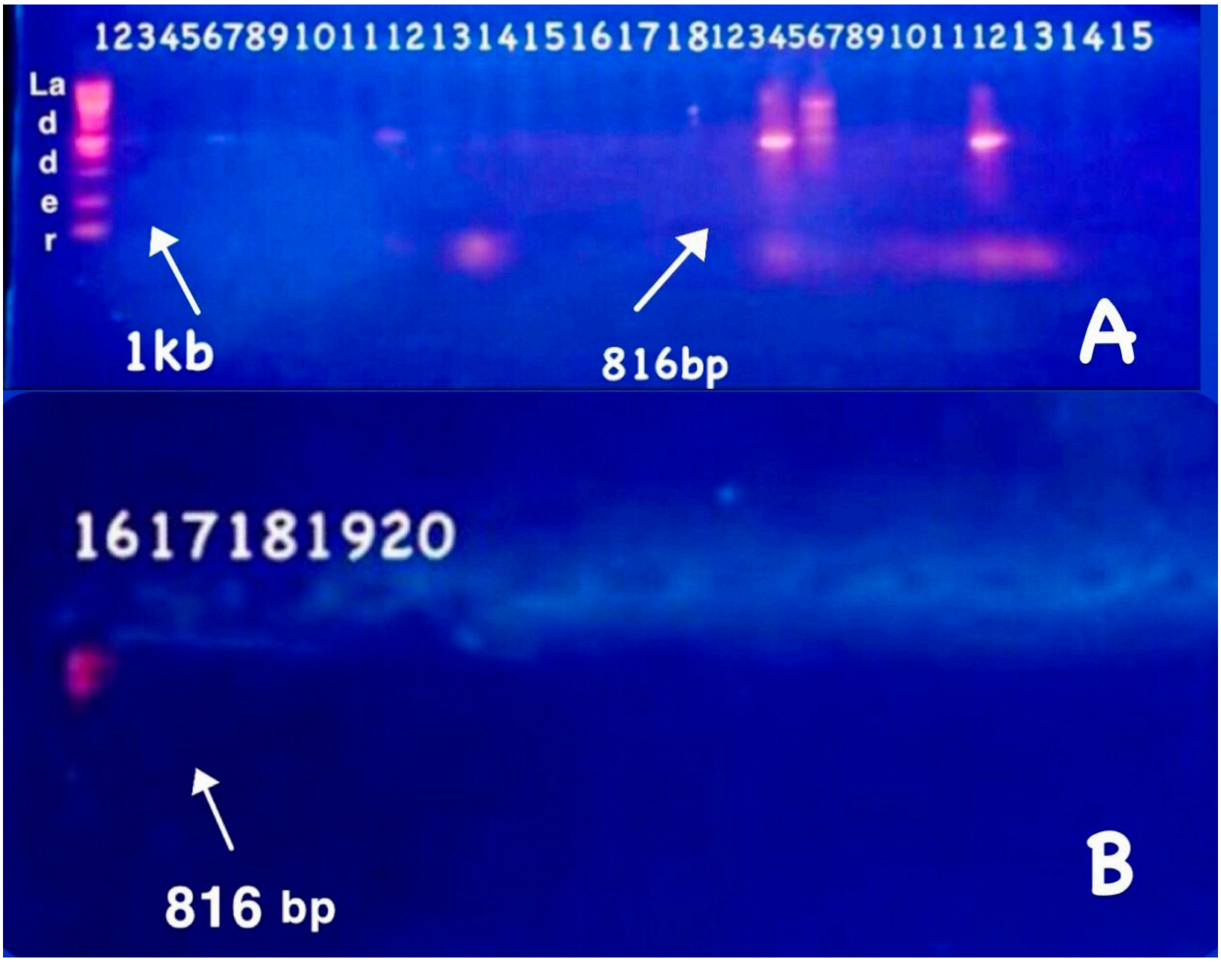

Figure S3: The OXA gene band of K. Pneumoniae generated for the ESBL sample in gel electrophoresis. A +B The OXA gene band of *K. pneumoniae* generated for the ESBL sample in gel electrophoresis. B- The OXA gene band of *K. pneumoniae* generated for the ESBL sample in gel electrophoresis. This figure shows samples from (South 1- 17) and (North 1-20).

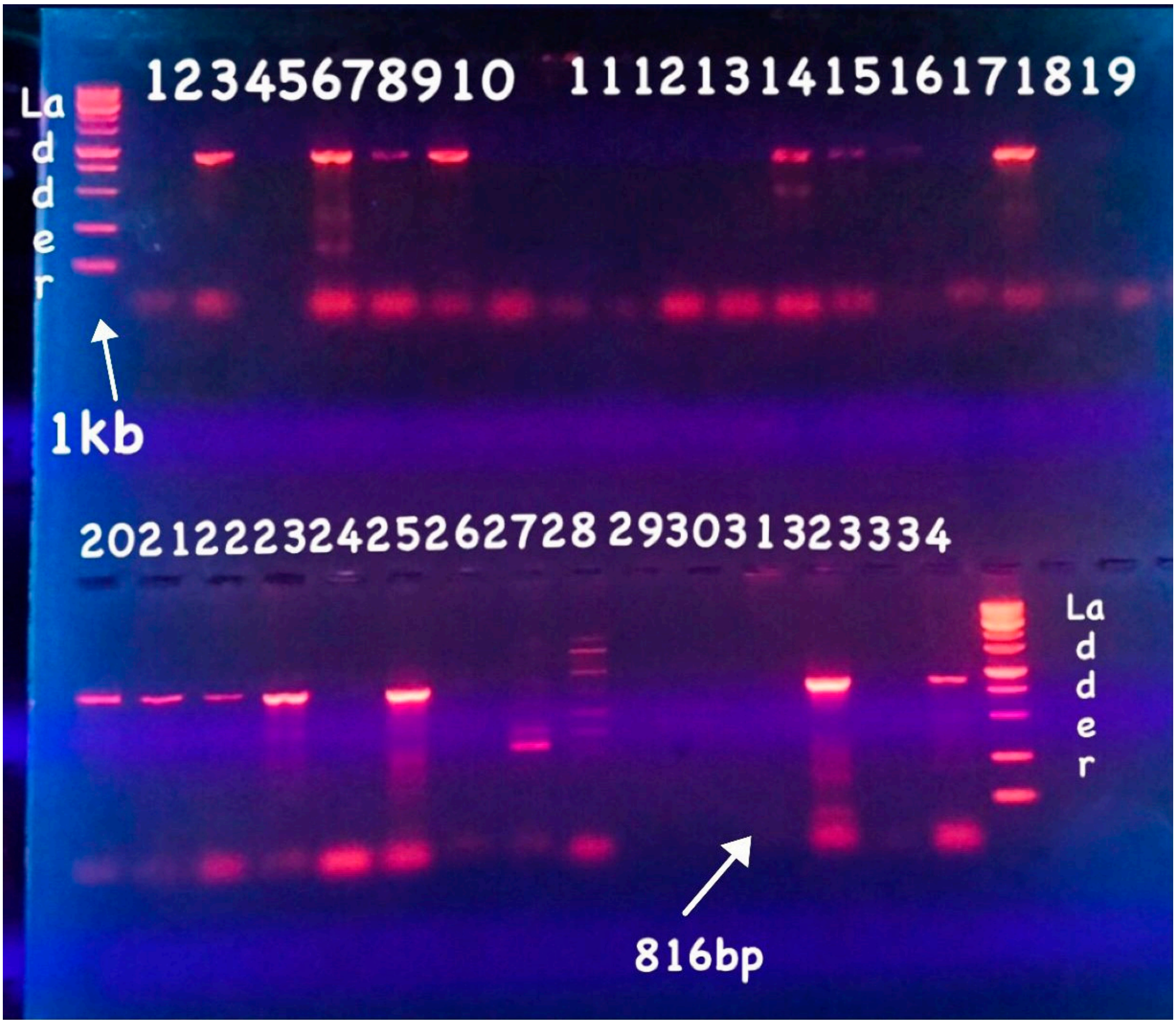

Figure S4: The OXA gene of *K. Pneumoniae* generated for ESBL sample in gel electrophoresis. This figure shows samples from (Middle 1-34).

OXA and CTX-M Genes:

Out of a total of 82 cases, 72 (87.8%) were included, and 10 (12.2%) were excluded across all variables tested, specifically for the prevalence of OXA and CTX-M genes. This pattern was consistent across location, hospital name, gender, and age for both OXA and

CTX-M genes, indicating a similar inclusion-to-exclusion ratio across these categories. Each variable demonstrated an 87.8% inclusion rate and a 12.2% exclusion rate, maintaining 100% consistency across all tests.

Table S1: Prevalence and Distribution of OXA and CTX-M Genes in K. Pneumoniae Isolates from UTI Patients Across Jordan.

	Included		Excluded		Total	
	N	Percent	N	Percent	N	Percent
Location * OXA gene	72	87.8%	10	12.2%	82	100.0%
Hospital name * OXA gene	72	87.8%	10	12.2%	82	100.0%
Gender * OXA gene	72	87.8%	10	12.2%	82	100.0%
Age * OXA gene	72	87.8%	10	12.2%	82	100.0%
Location * CTX-M gene	72	87.8%	10	12.2%	82	100.0%
Hospital name * CTX-M gene	72	87.8%	10	12.2%	82	100.0%
Gender * CTX-M gene	72	87.8%	10	12.2%	82	100.0%
Age * CTX-M gene	72	87.8%	10	12.2%	82	100.0%

The analysis of the OXA gene distribution in K. pneumoniae isolates showed that positive cases had slightly higher mean values across location and hospital name, while gender and age differences were minimal. Negative cases demonstrated lower variation in these factors. Overall, the presence of the OXA gene varied slightly by location and hospital, with consistent values for gender and age across both positive and negative groups.

The ANOVA results demonstrate that location and hospital name significantly impact the distribution of the OXA gene ($p = 0.019$), while gender and age do not show significant effects ($p = 0.820$ and $p = 0.682$, respectively).

Table S2: Distribution Patterns of The OXA Gene in K. Pneumoniae Isolates by Location, Hospital, Gender, and Age.

OXA gene		Location	Hospital name	Gender	Age
Negative	Mean	2.06	2.06	1.15	2.83
	N	48	48	48	48
	Std. Deviation	.810	.810	.357	1.209
Positive	Mean	2.54	2.54	1.17	2.71
	N	24	24	24	24
	Std. Deviation	.779	.779	.381	1.233
Total	Mean	2.22	2.22	1.15	2.79
	N	72	72	72	72
	Std. Deviation	.826	.826	.362	1.210

Table S3: Impact of Location, Hospital Name, Gender, And Age on the Distribution of The OXA Gene in K. Pneumoniae Isolates.

			Mean Square	F	Sig.
Location * OXA gene	Between Groups	(Combined)	3.674	5.744	.019
	Within Groups		.640		
	Total				
Hospital name * OXA gene	Between Groups	(Combined)	3.674	5.744	.019
	Within Groups		.640		
	Total				
Gender * OXA gene	Between Groups	(Combined)	.007	.052	.820
	Within Groups		.133		
	Total				
Age * OXA gene	Between Groups	(Combined)	.250	.169	.682
	Within Groups		1.480		
	Total				

The t-test analysis shows significant differences in the CTX-M gene distribution for hospital name and gender ($p = 0.019$), while location and age do not exhibit significant

differences (p = 0.618 and p = 0.820, respectively). The mean differences for significant factors were -0.479, indicating a notable variance in gene distribution across these categories.

Table S4: Assessment of CTX-M Gene Distribution Across Location, Hospital, Name, Gender, and Age.

		Sig. (2-tailed)	Mean Difference	Std. Error Difference
CTX-M gene	Equal variances assumed	.618	.063	.125
	Equal variances not assumed	.617	.063	.124
Location	Equal variances assumed	.019	-.479-	.200
	Equal variances not assumed	.019	-.479-	.197
Hospital name	Equal variances assumed	.019	-.479-	.200
	Equal variances not assumed	.019	-.479-	.197
Gender	Equal variances assumed	.820	-.021-	.091
	Equal variances not assumed	.824	-.021-	.093
Age	Equal variances assumed	.682	.125	.304
	Equal variances not assumed	.685	.125	.306

The 95% confidence intervals for the CTX-M gene distribution indicate significant mean differences for hospital name and gender (intervals do not include zero), while location and age show no significant differences (intervals do not include zero).

Table S5: Conference Interval Analysis of Mean Differences in CTX-M Gene Distribution by Location, Hospital Name, Gender, and Age.

	CTX-M gene	N	Mean	Std. Deviation	Std. Error Mean
Location	Negative	42	2.26	.828	.128
	Positive	30	2.17	.834	.152
Hospital name	Negative	42	2.26	.828	.128
	Positive	30	2.17	.834	.152
Gender	Negative	42	1.10	.297	.046
	Positive	30	1.23	.430	.079
Age	Negative	42	2.60	1.211	.187
	Positive	30	3.07	1.172	.214
OXA gene	Negative	42	.36	.485	.075
	Positive	30	.30	.466	.085

The data shows the distribution of OXA gene cases across three locations—south, north, and middle. Among the 72 total cases, 48 tested negatives for the OXA gene, with 14 cases in the south, 17 in the north, and another 17 in the middle. In contrast, of the 24 positive cases, only 4 were located in the south, 3 in the north, and 17 in the middle. This distribution highlights a noticeably higher concentration of OXA-positive cases in the middle location compared to the south and north.

Table S6: Geographical Distribution of OXA Gene Cases Across Regions.

		Location			
		South	North	Middle	Total
OXA gene	Negative	14	17	17	48
	Positive	4	3	17	24
Total		18	20	34	72

The distribution of CTX-M gene cases in the middle, north, and south is shown in the data. 42 of the 72 cases—10 from the south, 11 from the north, and 21 from the middle—were found to be negative for the CTX-M gene. Thirteen of the 30 positive instances, however, were found in the middle, nine in the north, and eight in the south. According to this distribution, the middle location has a noticeably greater concentration of CTX-M positive cases than the other two locations.

Table S7: Geographical Distribution of CTX-M Gene Cases Across Regions.

Count

		Location			Total
		South	North	Middle	
CTX-M gene	Negative	10	11	21	42
	Positive	8	9	13	30
Total		18	20	34	72
